# Targeting mTOR and Survivin Concurrently Potentiates Radiation Therapy in Renal Cell Carcinoma by Suppressing DNA Damage Repair and Amplifying Mitotic Catastrophe

**DOI:** 10.21203/rs.3.rs-3770403/v1

**Published:** 2023-12-23

**Authors:** Hari K. Rachamala, Vijay S. Madamsetty, Ramcharan S. Angom, Naga M. Nakka, Shamit Kumar Dutta, Enfeng Wang, Debabrata Mukhopadhyay, Krishnendu Pal

**Affiliations:** Mayo Clinic in Florida; Mayo Clinic in Florida; Mayo Clinic in Florida; Mayo Clinic in Florida; Mayo Clinic in Florida; Mayo Clinic in Florida; Mayo Clinic in Florida; Mayo Clinic in Florida

**Keywords:** Renal Cancer, Radiation Therapy, mTOR, Survivin, Mitotic catastrophe

## Abstract

**Background:**

Renal cell carcinoma (RCC) was historically considered to be less responsive to radiation therapy (RT) compared to other cancer indications. However, advancements in precision high-dose radiation delivery through single-fraction and multi-fraction stereotactic ablative radiotherapy (SABR) have led to better outcomes and reduced treatment-related toxicities, sparking renewed interest in using RT to treat RCC. Moreover, numerous studies have revealed that certain therapeutic agents including chemotherapies can increase the sensitivity of tumors to RT, leading to a growing interest in combining these treatments. Here, we developed a rational combination of two radiosensitizers in a tumor-targeted liposomal formulation for augmenting RT in RCC. The objective of this study is to assess the efficacy of a tumor-targeted liposomal formulation combining the mTOR inhibitor everolimus (E) with the survivin inhibitor YM155 (Y) in enhancing the sensitivity of RCC tumors to radiation.

**Experimental Design:**

We slightly modified our previously published tumor-targeted liposomal formulation to develop a rational combination of E and Y in a single liposomal formulation (EY-L) and assessed its efficacy in RCC cell lines in vitro and in RCC tumors in vivo. We further investigated how well EY-L sensitizes RCC cell lines and tumors toward radiation and explored the underlying mechanism of radiosensitization.

**Results:**

EY-L outperformed the corresponding single drug-loaded formulations E-L and Y-L in terms of containing primary tumor growth and improving survival in an immunocompetent syngeneic mouse model of RCC. EY-L also exhibited significantly higher sensitization of RCC cells towards radiation in vitro than E-L and Y-L. Additionally, EY-L sensitized RCC tumors towards radiation therapy in xenograft and murine RCC models. EY-L mediated induction of mitotic catastrophe via downregulation of multiple cell cycle checkpoints and DNA damage repair pathways could be responsible for the augmentation of radiation therapy.

**Conclusion:**

Taken together, our study demonstrated the efficacy of a strategic combination therapy in sensitizing RCC to radiation therapy via inhibition of DNA damage repair and a substantial increase in mitotic catastrophe. This combination therapy may find its use in the augmentation of radiation therapy during the treatment of RCC patients.

## BACKGROUND

Kidney cancer is one of the ten most prevalent cancers in the United States, ranking as the sixth and ninth most common cancer in men and women, respectively (1). In 2023, it is anticipated that around 81,800 new cases of kidney cancer will be diagnosed in the United States, resulting in 14,890 deaths (1). Renal cell carcinoma (RCC) accounts for approximately 90% of all kidney cancer cases (2). While early-stage RCC patients have a better prognosis, the survival rate for advanced-stage RCC patients is dismal, with a five-year survival rate of 12%–15% only (1). One-third of RCC patients present with widespread metastasis at diagnosis, and nearly half of the patients who undergo primary tumor resection develop distant metastasis (3). Existing therapies for advanced RCC, including chemotherapy, radiotherapy, and targeted therapies such as tyrosine kinase inhibitors (TKI), mammalian target of rapamycin (mTOR) inhibitors, or vascular endothelial growth factors (VEGF)-targeted therapies are unable to provide long-term survival benefits (4). Recently, immune checkpoint inhibitors (ICI) have been approved for the treatment of advanced RCC, either alone or in combination with TKI, following promising results in large Phase III trials (5–8). Nonetheless, alternative therapies are necessary for patients who suffer from severe side effects, experience disease progression after an initial positive response, or fail to respond altogether to ICI (9).

Apart from immunotherapy, radiation therapy (RT) is another effective curative treatment method for cancer (10). However, different types of cancer have varying degrees of resistance to RT, with RCC being known to have relatively higher resistance compared to other cancer types (11, 12). Cancer cells develop resistance to RT through various mechanisms, including DNA damage repair, cell cycle arrest, changes in oncogenic and tumor suppressor signaling pathways, tumor microenvironment (TME) remodeling, cancer stemness, and metabolic reprogramming (13). However, recent advancements in treatment planning, delivery techniques, immobilization strategies, image guidance, and computed tomography have substantially enhanced the effectiveness of RT. Assisted by modern computing power, single-fraction and multi-fraction stereotactic ablative radiotherapy (SABR) have achieved greater precision in delivering high-dose radiation, resulting in better treatment outcomes while minimizing treatment-related toxicities (14). Consequently, numerous clinical trials are currently investigating the effectiveness of SABR, either alone or in combination with other treatment modalities, as viable treatment options for RCC (12). However, combining SABR with agents that can override RCC’s intrinsic resistance to RT is more likely to improve therapeutic outcomes. Several studies have already demonstrated that certain therapeutic agents, including chemotherapy, can act as radiosensitizers, thereby prompting research studies combining RT with such agents (15).

Among the radiosensitizers, mTOR inhibitors such as everolimus enjoy distinct advantages over other chemotherapeutic agents since they also exert inherent antitumor and antiangiogenic properties in RCC (16, 17). Notably, mTOR inhibitors disrupt multiple mechanisms associated with radioresistance in cancer cells, including cancer stemness, metabolic pathways, DNA damage repair pathways, and various oncogenic pathways (18). Consequently, several clinical trials investigated the efficacy of combining RT with everolimus across various cancer types, including RCC (19–23). While this approach demonstrated efficacy in some patients, its overall clinical significance was compromised by dose-limiting toxicities (19, 24, 25).

Survivin expression has also been found to be associated with RT resistance, and genetic depletion or chemical inhibition of survivin has been shown to enhance radiosensitivity across various cancer types (26–30). Survivin is implicated in multiple RT resistance mechanisms including DNA damage repair, cell cycle, metabolic reprogramming, and stemness (31–33). YM155, a small imidazolium-based molecule, effectively inhibits the expression of survivin at both mRNA and protein levels and demonstrates significant antitumor efficacy and radiosensitizing activity in numerous animal models of cancer (30, 34). Notably, YM155 has shown the capacity to overcome resistance to mTOR inhibitors in renal and breast cancer (35, 36). Given these observations, we postulated that YM155 would synergize with everolimus in sensitizing RCC cells to RT. Interestingly, despite being tested in numerous clinical trials, YM155 has not yet received approval for clinical use (37). The lack of success in clinical trials may be attributed to its poor pharmacokinetic stability, as indicated by studies revealing a rapid decline in YM155 levels in both serum and tumors after completing treatment (38).

Combination therapies can surmount drug resistance, but often the ensuing increase in toxicity compels the discontinuation of therapy or dose reductions (39). To address this issue, target-specific drug delivery platforms are being explored with the capacity to deliver multiple drugs concurrently to tumors (40). Previously we developed a tumor-targeted liposomal formulation that shows promise in delivering multiple drugs to tumors effectively without eliciting toxicity in animal models (41, 42). Hence, we hypothesized that a similar tumor-targeted liposomal formulation combining everolimus with YM155 will have better efficacy and reduced systemic toxicity and will synergistically sensitize RCC tumors towards RT. The goal of this study is to determine whether this tumor-targeted liposomal formulation combining everolimus and YM155 inhibits growth in RCC tumors and at the same time sensitizes them to radiation therapy.

## METHODS

### Reagents

DOPC and DSPE-(PEG)2000-OMe were purchased from Avanti Polar Lipids and Nanosoft Polymers, respectively. Cholesterol was purchased from Sigma. TTP-conjugated lipopeptide was synthesized as described previously. Everolimus and YM155 were obtained from LC laboratories and MedChemExpress, respectively. Antibodies against mTOR, phospho-mTOR, p70S6K, phospho-p70S6K, survivin, ATM, PARP1, and β-actin were obtained from Cell Signaling Technology. ATR, CHk1, and Chk2 antibodies were obtained from Santa Cruz Biotechnology. CD3, CD8, and Ki67 antibodies were from Abcam, while CD45 antibody was from Biolegend.

### Cell Culture

786-O cell line was obtained from American Type Culture Collection (ATCC). Renca cell line was a kind gift from Dr. John A. Copland (Mayo Clinic). No authentication of the cell lines was done by the authors. 786-O cell line was maintained in Dulbecco’s Modified Eagle Medium (DMEM) and RPMI-1640 medium was used for maintaining Renca cell lines. Both the media were supplemented with 10% FBS and 1% penicillin–streptomycin (Invitrogen) and cells were cultured at 37°C in a humidified atmosphere with 5% CO_2_. Cells from 85%–90% confluent cultures were used in the experiments.

### Preparation and characterization of drug-loaded liposomes

A modified ethanol injection technique was employed to formulate the E-L, Y-L, or EY-L liposomes. Briefly, required amounts of DOPC (3.93 mg), Cholesterol (0.483 mg), DSPE-PEG(2000)-OMe (0.27 mg), and TTP-conjugated lipopeptide (0.22 mg) with everolimus (0.4 mg), and/or YM155 (0.8 mg) were dissolved in 400 μL ethanol and the solution was warmed at 65°C for 5 minutes. Subsequently, this ethanolic solution was slowly injected into 600 μL preheated milli-Q water at 65°C while continuously vortexing the mixture, resulting in the spontaneous formation of liposomes. Removal of unentrapped drugs and liposome characterization were performed as described previously (41, 42).

### In vitro cytotoxicity assay

Approximately, 5 × 10^3^ 786-O or Renca cells per well were seeded in 96-well plates and allowed to settle for 18–24 hours. Then, cells were treated with increasing concentrations of E-L, Y-L, and EY-L diluted in respective media and incubated for an additional 72 hours (n = 3 wells per concentration). Cell viability was determined with Celltiter 96 Aqueous One Solution Cell Proliferation Assay kit (Promega) as described previously (41, 42).

### Animals used in the study

Six- to eight-week-old SCID and Balb/c mice were obtained from in-house breeding and housed in the institutional animal facilities. All animal experiments were performed following the Association for Assessment and Accreditation of Laboratory Animal Care (AAALAC) guidelines under protocols approved by the Mayo Clinic Institutional Animal Care and Use Committee (IACUC).

### In vivo tumor regression experiment in subcutaneous Renca tumors:

The in vivo tumor regression efficacy of the drug-loaded liposomes was analyzed in syngeneic subcutaneous Renca tumors developed in Balb/c mice (n = 5 per treatment group). E-L (1.94 mg/kg E), Y-L (1.44 mg/kg Y), and EY-L (1.94 mg/kg E, 1.44 mg/kg Y) were intravenously administered twice a week for 4 weeks to mice bearing ~ 50 mm^3^ tumors. Tumors were measured weekly with calipers and tumor volumes were calculated using the formula: Volume = 0.5 × a × b^2^ where a and b are the longest and shortest diameter, respectively. Tumor growth curves were obtained by plotting tumor volumes against time. Finally, mice were sacrificed to harvest the tumors for immunohistochemistry.

### In vivo tumor regression experiment in orthotopic Renca tumors

We further analyzed the efficacy of EY-L in syngeneic orthotopic Renca tumors developed in Balb/c mice (n = 4 for control and n = 5 for EY-L treatment group). EY-L (1.94 mg/kg E, 1.44 mg/kg Y) was intravenously administered twice a week for 4 weeks to mice bearing orthotopic Renca tumors starting after 2 weeks of implantation. Tumor growth was monitored weekly by measuring bioluminescence in an IVIS Xenogen (Perkin Elmer). Tumor growth curves were obtained by plotting fold changes in bioluminescence from initial values against time. The survival was also analyzed by monitoring the IACUC-approved endpoint for each mouse.

### In vitro radiosensitivity experiments

For in vitro radiosensitivity, RCC cells were plated in 2 sets of 6 well plates and treated with PBS, E-L, Y-L, and EY-L for 48 hours. The sub-IC50 concentration of liposomes (0.01% for 786-O, 0.1% for Renca) was selected based on the results from the MTT assay to minimize cell death due to drug treatment only. One set of cells was then exposed to 2 Gy radiation at room temperature at a 3.9 Gy/min dose rate and a 160 kV tube voltage using an X-RAD 160 Irradiator (Precision X-Ray Inc., USA). Following irradiation, the cell samples were returned to a 5% CO_2_ incubator. Both irradiated and non-irradiated cells were then harvested and seeded in triplicates (100 cells/well) in 12-well plates in fresh culture media without drugs and allowed to grow for 10–14 days. Then, colonies were fixed with 4% formaldehyde and stained with 0.2% Crystal Violet solution, and colonies larger than 30 μm in diameter were counted. The surviving fraction for a particular treatment group was determined by dividing the plating efficiency of the irradiated cells by the plating efficiency of the corresponding unirradiated cells.

#### Immunoblot analysis:

Lysates were prepared from treated cells using NP-40 lysis buffer supplemented with a protease inhibitor cocktail. Protein concentrations of the lysates were measured by Bradford assay. Equal amounts of proteins from each sample were subjected to SDS-PAGE and transferred to polyvinyl difluoride membranes followed by immunoblotting with primary antibodies and respective secondary antibodies (1:10000). Enzyme-linked chemiluminescence was used to detect antibody-reactive bands in Chemidoc MP (Bio-Rad). Blots from the same experiments were used for presentation.

### In vivo radiosensitivity experiments

To evaluate the in vivo radiosensitization potential of EY-L in RCC tumors, we first developed subcutaneous 786-O xenografts by implanting 5 × 10^6^ cells into the right flanks of 6–8 weeks old SCID mice. When the tumors became palpable, twice-a-week EY-L (1.94 mg/kg E, 1.44 mg/kg Y) intravenous administrations were started and continued for 3 weeks. Two doses of focused single-beam 10 Gy radiation each were administered to the tumors on days 12 and 19 for mice belonging to the radiation-only (R) and combination group (EY-L + R). Radiation was administered at 2.9 Gy/min in an XRAD-SmART instrument (225 kV, 13 mA). Additionally, a separate group of mice (R-early) received two doses of focused 10 Gy radiation on days 5 and 12. This was done to ensure that their initial average tumor volume was similar to that of the EY-L + R group at the time of the first radiation dose. Treatment was stopped after three weeks, and tumor growth was monitored for another 3 weeks.

A similar experiment was conducted using subcutaneous Renca tumors developed in syngeneic Balb/c mice. Here, we only kept the R-Early group for the radiation-only treatment group for a more stringent comparison of the combination group with the radiation-only group. Treatment was discontinued after three weeks, and tumor growth was closely monitored until an IACUC-approved endpoint was reached for each mouse. Given the distinct endpoints for each mouse, we refrained from using the tumor tissues from this particular experiment. Instead, we conducted a similar experiment with another group of tumor-bearing mice and concluded it after 21 days (i.e., two days following the final radiation dose). This allowed us to harvest tumors for immunohistochemistry analysis, focusing on potential alterations in immune-cell infiltrations within the tumor microenvironment resulting from the treatment. Here, the radiation treatments were performed on the same days (i.e., days 12 and 19) in both the combination group and the radiation-only group to keep the timeline the same between radiation and harvesting of tumors in these two groups.

#### Immunohistochemistry:

Tumors were harvested and fixed in neutral buffered 10% formalin at room temperature for 24 hours. Then they were embedded in paraffin and 5 μm thick sections were cut for preparing slides. Hematoxylin and eosin (H&E), Ki67 (1:1000), CD45 (1:1000), CD3 (1:1000), and CD8 (1: 1000) staining were performed in deparaffinized slides as applicable following the manufacturer’s instructions (DAB 150; Millipore). Slides were stained with stable diaminobenzidine and counterstained with hematoxylin. Finally, slides were digitized using an Aperio AT2 slide scanner (Leica) and analyzed using ImageScope software (Leica).

#### Immunocytochemistry:

Tumors were harvested and fresh frozen in OCT medium where applicable. Then, 5 μm thick sections were cut from these fresh frozen tumors for preparing slides. Pericentrin (1:1000) staining was performed in these fresh frozen sections. Slides were stained with Alexa-Fluor-670 conjugated secondary antibody. Finally, slides were mounted in Vectashield mounting medium containing DAPI and imaged using an LSM 780 Confocal microscope and analyzed.

### Statistical analyses

Microsoft Excel and GraphPad Prism were used for data analyses. One-way ANOVA followed by Tukey’s post-hoc analysis or double-sided unpaired two-tailed t-test was utilized to determine the probability of significant differences between treatment groups where applicable. For tumor growth curves, the endpoint tumor volumes were compared for statistically significant differences among each other using a double-sided unpaired two-tailed t-test where applicable. Statistical significance was defined as p < 0.05 (*), p < 0.01 (**), p < 0.001 (***), and p < 0.0001 (****) respectively. Error bars are indicative of calculated SD values.

## RESULTS

### EY-L is a homogeneous, positively charged nanoformulation

The amount of lipid and drug components of the drug-loaded liposomes (E-L, Y-L, and EY-L) are reported in **Supplementary Table S1** along with drug loading efficiency (DLE) and encapsulation efficiency (EE) values. The initial amounts of Everolimus and YM155 used during the preparation of liposomes were 0.4 mg and 0.8 mg per 1 mL of liposomes respectively. Everolimus, being a highly water-insoluble lipophilic drug, displayed an EE of 98.19% ± 2.13% in E-L and 96.73% ± 2.01% in EY-L due to its nearly complete incorporation in the liposome bilayer. YM155 displayed only 37.14% ± 1.70% EE in Y-L and 36.05% ± 2.35% in EY-L due to its hydrophilic nature. The DLE values for Everolimus in E-L and YM155 in Y-L were 7.42% ± 0.16% and 5.71% ± 0.26% respectively. On the other hand, The DLE values for Everolimus and YM155 in EY-L were 6.94% ± 0.14% and 5.17% ± 0.34% respectively. The EE values in dual drug-loaded liposomes (EY-L) did not show statistically significant alterations from the single drug-loaded ones, albeit they were slightly lower. Plausibly, the distinct spatial distribution of Everolimus and YM155 inside the liposomes is not affecting their individual encapsulation efficiencies. However, the DLE values of the EY-L differed more from E-L or Y-L due to the increased total weight of the EY-L liposomes containing both drugs over E-L or Y-L liposomes containing a single drug.

The average hydrodynamic size, polydispersity index (PDI), and zeta potential of E-L, Y-L, and EY-L are consolidated in **Supplementary Table S2**. The hydrodynamic diameters of E-L, Y-L, and EY-L were 62.15 nm ± 0.40 nm, 67.55 nm ± 0.24 nm, and 67.15 nm ± 0.31 nm, respectively. All the liposomal formulations had an average size of less than 100 nm which is suitable for better penetration through the tumor microenvironment (43). The polydispersity indices of E-L, Y-L, and EY-L were 0.178 ± 0.015, 0.195 ± 0.007, and 0.205 ± 0.01, respectively, suggesting excellent uniformity of the liposomes. The zeta potentials of E-L, Y-L, and EY-L were 10.23 mV ± 2.4 mV, 32.7 mV ± 4 mV, and 37.5 mV ± 3.3 mV, respectively. A positive zeta potential indicates the stability of the liposomal suspension as well as stronger interaction with negatively charged cell membranes. Since all these liposomes were positively charged suggesting these formulations to be stable and efficient in cellular uptake (44).

### EY-L shows a robust antiproliferative effect in RCC cells in vitro

**Figure 1:**
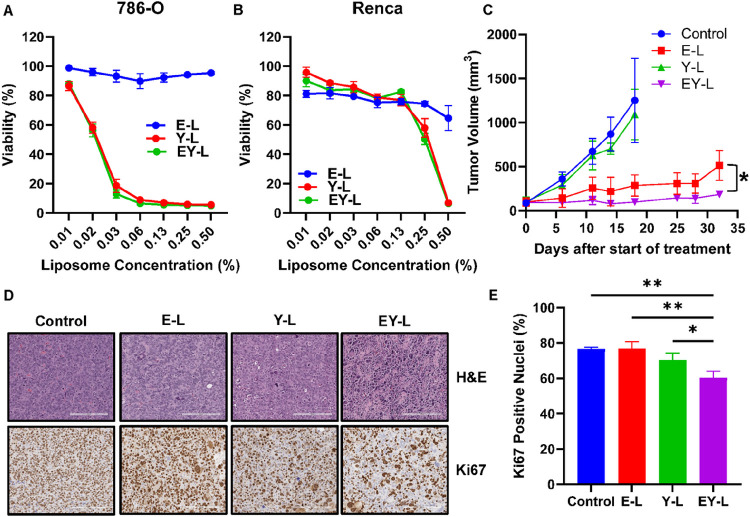
EY-L demonstrates a remarkable antiproliferative effect in RCC in vitro and in vivo. MTS assay in 786-O (A) and Renca (B) cells treated with increasing concentrations of E-L, Y-L, or EY-L for 72 hours (n=4 wells per treatment condition). Interestingly, E-L did not show a noticeable effect in vitro whereas Y-L and EY-L show almost similar curves, which suggests that YM-155 is solely responsible for the antiproliferative effect of EY-L in RCC, at least in vitro. Here, 1% liposome is equivalent to ~4.1 μM (in E-L) or ~4.04 μM (in EY-L) everolimus, and ~6.7 μM (in Y-L) or ~6.51 μM (in EY-L) YM155. (C) Approximately 1 × 10^6^ Renca cells were injected subcutaneously in 6–8 weeks-old female Balb/c mice. When the average size of tumors reached 100 mm^3^, twice a week i.v. treatment started with E-L (1.94 mg/kg E), Y-L (1.44 mg/kg Y), or EY-L (1.94 mg/kg E, 1.44 mg/kg Y) (n=5 mice per group). The treatment was continued for 4 weeks, and tumor growth was monitored weekly. Both E-L and EY-L showed excellent inhibition of tumor growth, but EY-L was the most effective. Surprisingly, Y-L did not show any noticeable tumor growth inhibition. * denotes p<0.05. (D) Representative images of H&E and Ki67 stained tumor sections from the above experiment. EY-L showed noticeable reductions in Ki67 staining. Bar length = 200 μm. (E) Quantitation of Ki67-positive nuclei in tumor sections. EY-L showed significant reductions in Ki67-positive nuclei compared to all other groups. * p<0.05, ** p<0.01, *** p<0.001.

Following characterization, we then proceeded to assess the in vitro cytotoxicities of the drug-loaded liposomal formulations in 786-O and Renca cells. Interestingly, E-L did not show significant cytotoxicity at the concentrations tested in either of the cells whereas both Y-L and EY-L showed similar cytotoxicity in both cases (Fig. 1A–B). 786-O cells were more sensitive towards Y-L or EY-L treatment than Renca, the IC50 values being more than tenfold less in 786-O cells (IC50 ~ 0.022% liposome) than in Renca cells (IC50 ~ 0.3% liposome). Here, 1% liposome is equivalent to ~ 4.1 μM (in E-L) or ~ 4.04 μM (in EY-L) everolimus, and ~ 6.7 μM (in Y-L) or ~ 6.51 μM (in EY-L) YM155.

### EY-L demonstrates superior inhibition of mTOR and survivin over E-L and Y-L, respectively

Western blot experiments demonstrate that EY-L was superior to E-L and Y-L in inhibiting phosphorylation of p70S6K (downstream of mTOR) and survivin expression, respectively **(Supplementary Fig. S1)**. This suggests that Everolimus and YM155 act synergistically to augment each other’s function when combined in a single formulation. Interestingly, the same amount of Everolimus alone (as E-L) was not able to inhibit phosphorylations of p70S6K in any of the cells. Y-L was effective at reducing survivin expression in 786-O cells only, but not in Renca cells. In contrast, EY-L was equally effective in inhibiting p70S6K phosphorylation and survivin expression in both cell lines.

### EY-L demonstrates a strong antitumor effect in a subcutaneous syngeneic murine RCC model

Inspired by the superior in vitro efficacy of EY-L, we proceeded to analyze the in vivo efficacy of the drug-loaded liposomes in a highly aggressive syngeneic mouse RCC model developed by subcutaneous implantation of Renca cells in immune-competent Balb/c mice. Both E-L and EY-L displayed remarkable tumor growth inhibition throughout the study, EY-L being the most effective treatment group (Fig. 1C). The individual tumor growth curves from this experiment are provided in **Supplementary Figure S2.** Interestingly, YM-155 did not show any visible tumor growth inhibition as a single liposomal formulation (Y-L) in this experiment but augmented the efficacy of everolimus when combined in the same liposomal formulation (EY-L). The H&E and Ki67 staining of the tumor sections demonstrates strong antiproliferative activity in EY-L-treated tumors (Fig. 1D–E).

### EY-L impedes tumor growth in an orthotopic syngeneic murine RCC model

**Figure 2:**
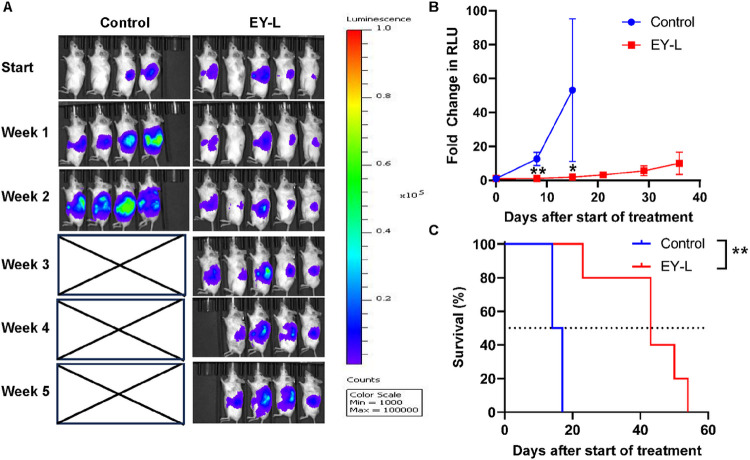
EY-L demonstrates a remarkable antitumor effect in an orthotopic syngeneic mouse model of RCC. (A) Approximately 1 × 10^5^ luciferase-transfected Renca cells were injected orthotopically into the left kidneys of 6–8 weeks-old female Balb/c mice. After 14 days, twice a week i.v. treatment started with EY-L (1.94 mg/kg E, 1.44 mg/kg Y) (n=4 for the control group, n=5 for EY-L group). The treatment was continued for 4 weeks, and tumor growth was monitored weekly by bioluminescence imaging. EY-L showed excellent inhibition of tumor growth as measured by reductions in the bioluminescence signals. The control mice reached the endpoint due to aggressive tumor growth after 2 weeks of starting treatment. (B) Tumor growth curves plotted as a fold change in RLU from initial measurements showed strong inhibition of tumor growth due to EY-L treatment. * and ** denote p<0.05 and p<0.01, respectively. (C) EY-L improved median overall survival compared to the control group (43 vs 15) as well. ** denotes p<0.01.

We further tested the efficacy of EY-L in an orthotopic syngeneic mouse ccRCC model developed by subcapsular implantation of luciferase-labeled Renca cells in immune-competent Balb/c mice. Since EY-L was the most effective in the previous experiment, we did not include E-L or Y-L in this experiment or further in vivo experiments. EY-L showed significant tumor growth inhibition (Fig. 2A–B) and enhanced median survival (Fig. 2C) compared to the control group in this model. The individual tumor growth curves from this experiment are provided in **Supplementary Figure S3.**

### EY-L sensitizes RCC cells toward radiation in vitro

**Figure 3:**
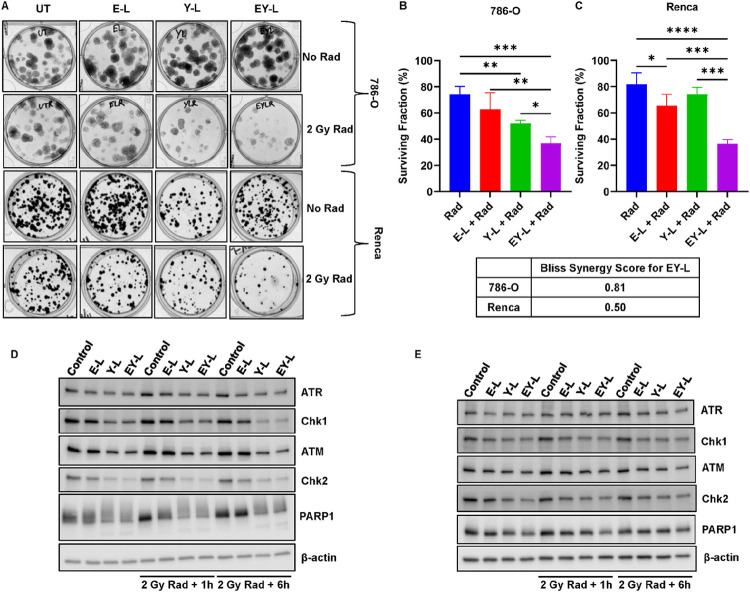
EY-L sensitizes RCC cells toward radiation in vitro by inhibiting cell cycle checkpoints and DNA damage repair. A) 786-O and Renca cells were treated for sub-IC50 concentrations of E-L, Y-L, and EY-L (0.01% liposomes for 786-O, 0.125% liposomes for Renca) for 48 hours followed by exposure to 2 Gy radiation. A ‘no radiation’ control was included for each of the treatment groups. Cells were then harvested and re-seeded in 12 well plates at a concentration of 100 cells/well. Colonies were allowed to grow for 14 days followed by fixation with 4% paraformaldehyde and staining with 0.2% crystal violet solution. Representative images of the colonies were included. (B-C) Colonies greater than 50 cells were counted under a microscope and surviving fractions were determined. EY-L treatment led to the highest reduction in the surviving fraction in both cell lines. Bliss synergy scores were 0.81 and 0.50 for 786-O (B) and Renca (C) cells, respectively, signifying a moderate to strong synergistic effect of EY-L. * p<0.05, ** p<0.01, *** p<0.001, **** p<0.0001. (D) 786-O and (E) Renca cells were treated for sub-IC50 concentrations of E-L, Y-L, and EY-L (0.01% liposomes for 786-O, 0.125% liposomes for Renca) for 48 hours followed by exposure to 2 Gy radiation. A ‘no radiation’ control was included for each of the treatment groups. Cells were then harvested and lysed, and Western Blot analysis was employed to determine alterations in expressions of various DNA damage repair proteins were analyzed. EY-L showed strong inhibition of ATR, Chk1, ATM, Chk2, and PARP1.

Since both E and Y individually had been shown to increase the sensitivity of different cancer cells toward radiation, we investigated if there is any synergistic effect of EY-L in the radiosensitization of RCC cells in vitro over E-L or Y-L by performing colony formation assay. We used both 786-O and Renca cells in this experiment. 786-O cells formed dispersed-type colonies with diffused staining whereas Renca cells formed well-defined colonies with good staining. Nonetheless, the EY-L treated group led to the lowest surviving fraction post-radiation than the other treatment groups including the control, E-L, or Y-L (Fig. 3A–C). The Bliss synergy scores for the radiosensitization of EY-L over E-L and Y-L were 0.81 and 0.50 for 786-O and Renca, respectively, suggesting a moderate-to-strong synergistic effect of the combination therapy.

### EY-L inhibits multiple DNA damage repair mechanisms

Efficient DNA damage repair mechanisms are required to alleviate the harmful effects of radiation. These pathways are typically exploited by various cancer cells to maintain their radioresistant nature. Some of the crucial proteins involved in DNA damage repair include PARP1 (widely recognized as a first-line responder molecule in DNA damage response), ATM/Chk2 (double-stranded break repair), and ATR/Chk1 single-stranded break repair). Not surprisingly, EY-L was highly effective and in most cases was better than E-L or Y-L in reducing the expressions of these proteins, even subduing any increase post-radiation in some instances (Fig. 3D–E).

### EY-L sensitizes RCC xenograft tumors toward radiation in vivo

**Figure 4:**
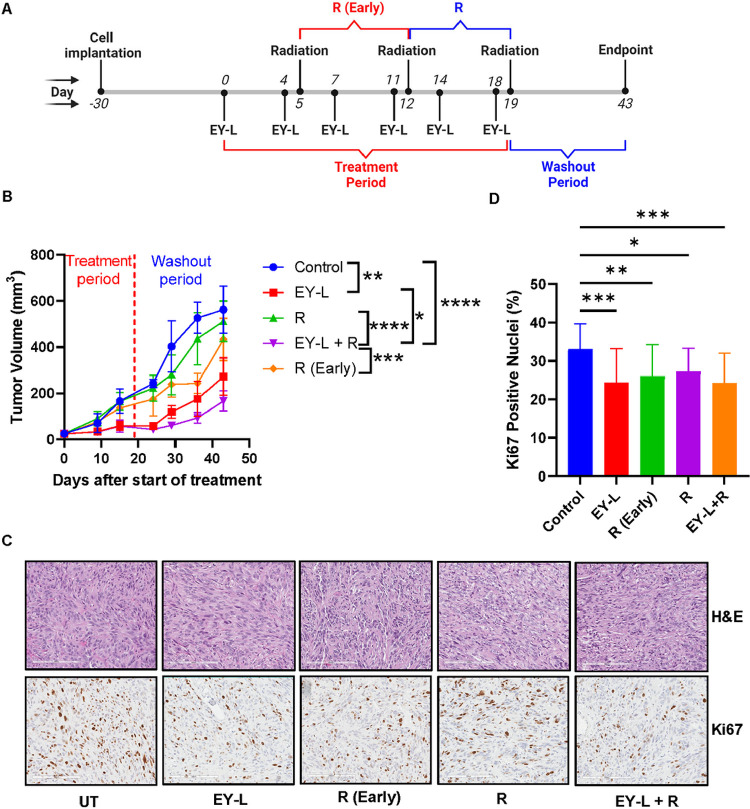
EY-L augments radiation therapy in an RCC xenograft model. (A) Approximately 5 × 10^6^ 786-O cells were injected subcutaneously in the right flanks of 6–8 weeks-old female SCID mice. After 30 days, twice a week i.v. treatment started with EY-L (1.94 mg/kg E, 1.44 mg/kg Y) (n=4 for the control group, n=5 for EY-L group) and continued for 3 weeks. Two doses of focused 10 Gy radiation each were administered to the tumors on days 12 and 19 for mice belonging to the radiation-only (R) and combination group (EY-L + R). Additionally, a separate group of mice (R-early) received two doses of focused 10 Gy radiation on days 5 and 12. Treatment was stopped after 3 weeks, but weekly tumor growth monitoring was continued for an additional 3 weeks of washout period. The EY-L + R treatment group showed the highest inhibition of tumor growth. * p<0.05, ** p<0.01, *** p<0.001, **** p<0.0001. (C) Representative images of H&E and Ki67 stained tumor sections from the above experiment. All the treatment groups showed noticeable reductions in Ki67 staining from the control group. Bar length = 200 μm. (D) Quantitation of Ki67-positive nuclei in tumor sections. All the treatment groups showed significant reductions in Ki67-positive nuclei compared to the control groups but were not significantly different among themselves. This might be due to the 3-week-long washout period. * p<0.05, ** p<0.01, *** p<0.001.

Inspired by the observed results from the in vitro radiosensitivity experiments and Western Blot analysis, we then proceeded to evaluate the in vivo radiosensitivity of EY-L. We first used subcutaneous 786-O xenografts developed in SCID mice to evaluate the in vivo radiosensitization potential of EY-L in the absence of any additional effects due to the immune system. We evaluated only EY-L in this experiment since it was superior to E-L and Y-L in vitro. The experiment timeline is provided in Fig. 4A. Treatment was stopped after three weeks (treatment period), and tumor growth monitoring was continued for another 3 weeks of washout period. As it is clear from the growth curve, the starting tumor volume of the R group was higher than that of the EY-L + R group whereas they are more or less similar between R-early and EY-L + R groups (Fig. 4B). The individual tumor growth curves from this experiment are provided in **Supplementary Figure S4**. Interestingly R (Early) group showed an initial difference from the R group due to early exposure to radiation but after 6 weeks there was no significant difference between them. EY-L + R group showed significant impedance in tumor growth compared to all other groups, suggesting the augmentation of radiation therapy by EY-L. We performed immunohistochemistry from the FFPE tumor tissues obtained after the endpoint. Interestingly, we did not see any significant difference in Ki67 staining among EY-L, R−, EY-L + R, and R (Early) groups although all of them were significantly lower than the control group (Fig. 4C–D). We believe this may be due to the waning of treatment-induced effects during an additional 21 days in the washout period.

### EY-L sensitizes syngeneic RCC tumors toward radiation in vivo

**Figure 5:**
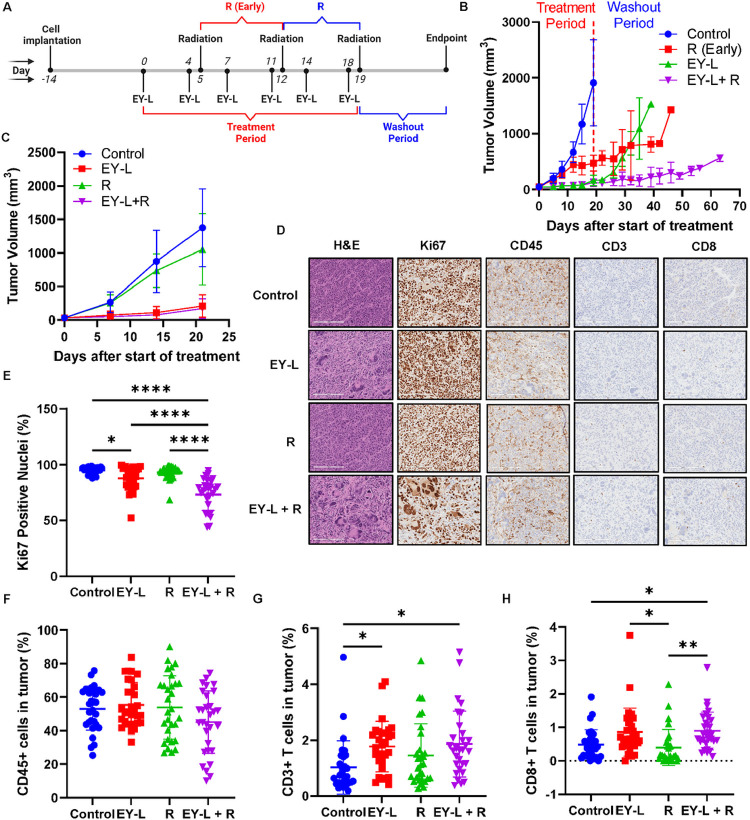
EY-L augments radiation therapy in a murine syngeneic RCC model. (A) Approximately 1 × 10^6^ Renca cells were injected subcutaneously in the right flanks of 6–8 weeks-old female Balb/c mice. After 14 days, twice a week EY-L (1.94 mg/kg E, 1.44 mg/kg Y, i.v.) treatment started (n=5 mice per group) and continued for 3 weeks. Two doses of focused 10 Gy radiation each were administered to the tumors on days 12 and 19 for mice belonging to the combination group (EY-L + R). The R (Early) group received two doses of focused 10 Gy radiation on days 5 and 12. Here we included only the R (Early) group based on the results obtained from the 786-O experiment for a stringent comparison. Treatment was stopped after 3 weeks, but twice-a-week tumor growth monitoring was continued throughout the washout period until the endpoint. The EY-L + R treatment group showed the highest inhibition of tumor growth. (C) A similar experiment was performed but was stopped 2 days after the final dose of radiation to harvest the tumors for immunohistochemistry. Here, the R (Early) group was replaced with the regular R group to keep the washout period the same between treatments. (D) Representative images of H&E, Ki67, CD45, CD3, and CD8 stained tumor sections from the above experiment. Bar length = 200 μm. (E) Quantitation of Ki67-positive nuclei in tumor sections (n=30, 10 visual fields each from 3 sections per group). The EY-L + R treatment group showed a significant reduction in Ki67-positive nuclei compared to all the other groups. * p<0.05, **** p<0.0001. (F-H) Quantitation of CD45+ (F), CD3+ (G), and CD8+ (H) cells in tumor sections (n=30, 10 visual fields each from 3 sections per group). No significant changes in CD45+ cells were observed, but the EY-L + R group showed the highest CD3+ and CD8+ staining suggesting greater infiltration of CD3+ and CD8+ T cells in the tumor. * p<0.05, ** p<0.01.

A similar experiment was performed in subcutaneous Renca tumors developed in syngeneic Balb/c mice to assess if the immune system plays any additional role in EY-L mediated radiosensitization. Here, we only kept the R (Early) group for the radiation-only treatment group for a more stringent comparison of the efficacy of the combination group with the radiation-only group. A similar experimental timeline as the above experiment was followed (Fig. 5A). Treatment was stopped after 3 weeks, and tumor growth was monitored until an IACUC-approved endpoint was reached for each mouse. As anticipated, EY-L + R treatment led to a noticeable inhibition of tumor progression compared to the control, EY-L, or R (Early) groups (Fig. 5B). The individual tumor growth curves from this experiment are provided in **Supplementary Figure S5**.

Based on our experience with the immunohistochemistry results in 786-O xenografts, we did not use the tumor tissues from the above experiment since there is a long washout period which may have reduced any therapy-induced effects in tumor tissues. Hence, we repeated the experiment in another set of tumor-bearing mice and stopped the experiment after 21 days (i.e., 2 days after the final radiation dose) to harvest tumors for immunohistochemistry to analyze any alterations in immune-cell infiltrations in the tumor microenvironment due to treatment (Fig. 5C). The radiation dosing schedules were kept same between R and EY-L + R (Day 12 and Day 19) in this experiment to remove any disparity in treatment-induced alterations in endpoint immunohistochemistry due to different dosing schedules and washout periods. The individual tumor growth curves from this experiment are provided in **Supplementary Figure S6**. Immunohistochemistry was performed on FFPE tumor tissue sections for H&E, Ki67, CD45, CD3, and CD8 (Fig. 5D). The quantification of Ki67, CD45, CD3, and CD8 staining was performed as well (Fig. 5E–H). The EY-L + R group showed significantly lower Ki67 positivity among all the groups (Fig. 5E). CD45 staining was not significantly affected among the treatment groups, although the EY-L + R group showed slightly lower abundance (Fig. 5F). CD3 + T cells were significantly higher in both EY-L and EY-L + R treatment groups compared to the control group (Fig. 5G). Interestingly, CD8 + T cells in both EY-L and EY-L + R treatment groups were significantly higher than control or R groups (Fig. 5H). However, no significant difference was observed between the EY-L and EY-L + R groups. Nonetheless, this experiment suggests that there is some additional effect of the immune system in EY-L mediated radiosensitization of the Renca tumors.

### EY-L induced mitotic catastrophe in RCC tumors which is aggravated by radiation exposure

**Figure 6:**
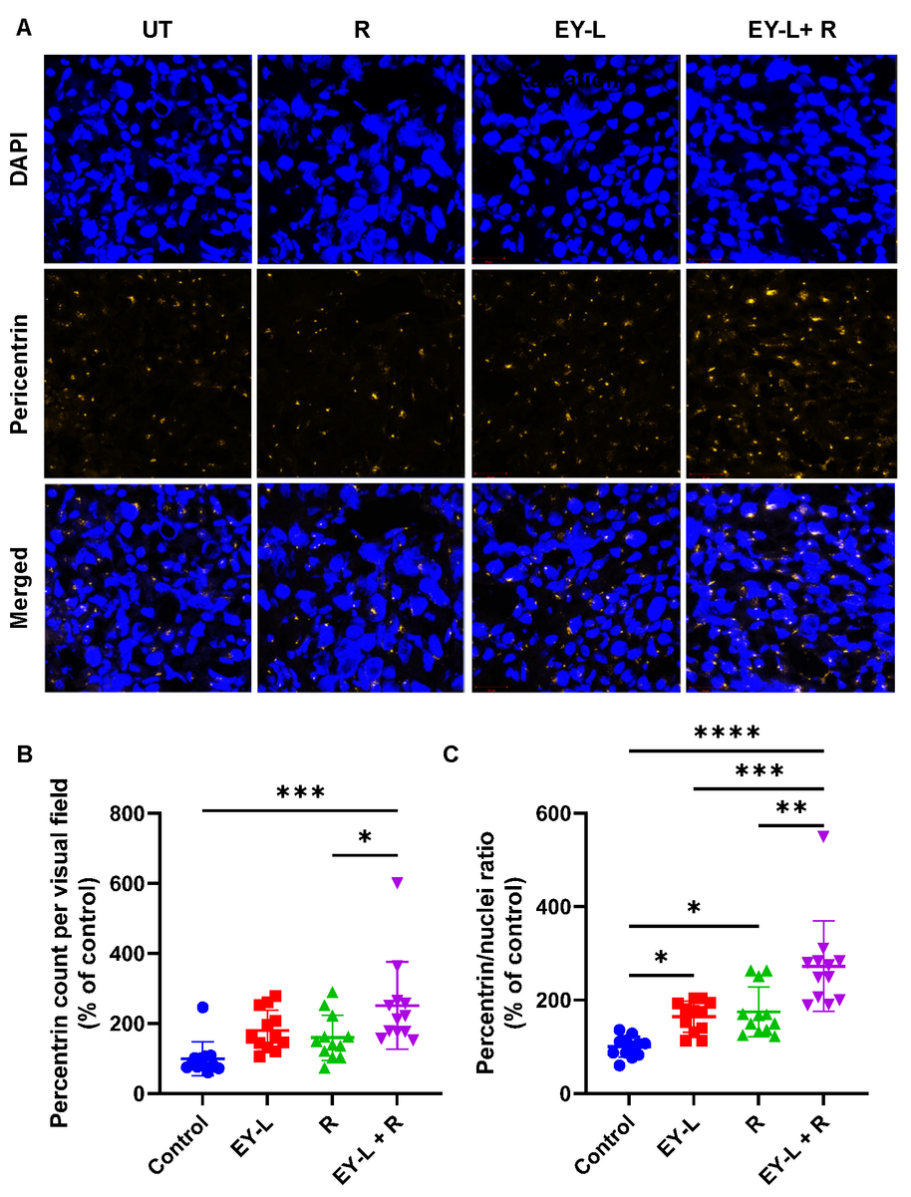
EY-L induces mitotic catastrophe in RCC tumors which is further enhanced by radiation therapy. (A). Representative confocal images of fresh frozen tumor sections from the above experiment stained for DAPI (top panel) and pericentrin (middle panel). The bottom panel shows the merged images. Magnification = 40X. (B-C) Plots showing the pericentrin count per visual field (B) or pericentrin/nuclei ratio (C). Individually, the EY-L and R groups showed higher values than the control, but EY-L + R demonstrated the highest pericentrin count or pericentrin/nuclei ratio. A higher pericentrin/nuclei ratio indicates a higher incidence of mitotic catastrophe. * p<0.05, ** p<0.01, *** p<0.001, **** p<0.0001.

The H&E staining of the tumor tissue sections obtained from the above experiment showed the presence of several multinucleated cells in the EY-L and EY-L + R treated tumors, the abundance being higher in the combination group (Fig. 5D). Giant multinucleated cells characterized by missegregated and uncondensed chromosomes are often the morphological markers of mitotic catastrophe. Radiation or other DNA-damaging treatment-induced centrosome amplification and subsequent formation of multipolar mitotic spindles are potential prerequisites of mitotic catastrophe. The pericentrin staining of fresh frozen tumor sections (Fig. 6A) from the above experiment showed a significant increase in pericentrin count in EY-L + R treated tumors than control or radiation-only tumors, but not EY-L treated tumors (Fig. 6B). However, the pericentrin/nuclei ratio, which is a closer estimate of centrosomes per cell, showed a significant increase in the EY-L + R group compared to all other groups (Fig. 6C) suggesting a significantly higher incidence of mitotic catastrophe in the EY-L + R group.

## DISCUSSION

The primary objective of RT in radiation oncology is to hinder the proliferation of cancer cells and ultimately eliminate them. RT employs various mechanisms to achieve this, including, apoptosis, autophagy, mitotic death (or mitotic catastrophe), necrosis, and senescence (45). However, given that radiation can harm both cancerous and healthy cells, the focus of RT is to maximize the radiation dose directed at the tumor while minimizing exposure to adjacent normal cells or those in the path of the radiation. Advanced technologies employed in RT delivery such as SBRT facilitate the administration of a maximum radiation dose to the tumor while sparing healthy tissues (14).

Another strategy to enhance radiation therapy treatment outcomes involves the use of radiosensitizers for radiosensitization of cancer cells (15). Radiosensitization is a process aimed at heightening the vulnerability of cancer cells to radiation-induced damage, while simultaneously minimizing potential harm to the adjacent healthy tissues. Radiosensitizers can affect cancer cells in various ways including increasing ROS within the cancer cells, inhibiting DNA repair mechanisms, modifying the tumor microenvironment, and targeting specific molecular pathways or proteins involved in cell survival and radiation resistance (46). In recent years, there has been a substantial surge in interest regarding the use of radiosensitizers to augment the efficacy of radiotherapy. Radiosensitizers can be categorized into three main groups based on their composition: small molecules, macromolecules, and nanomaterials (47). Radiosensitizers being evaluated in various clinical trials include Cisplatin, Gemcitabine, Olaparib, Paclitaxel, Temozolomide, Cetuximab, noble metal nanoparticles, and heavy metal nanoparticles (47).

We included everolimus and YM155, inhibitors of mTOR and survivin, respectively, as radiosensitizers in the present study. The selection of this combination was partly rationalized based on the findings of a couple of previous studies demonstrating that YM155 was able to overcome resistance to mTOR inhibitors in renal and breast cancer (35, 36). The result obtained from the tumor growth inhibition study in a subcutaneous murine RCC model further corroborated these observations (Fig. 1). EY-L was effective in impeding tumor growth and enhancing survival in orthotopic tumors as well (Fig. 2).

Additionally, both mTOR and survivin are implicated in cell proliferation, survival, and DNA damage response pathways, which are responsible for imparting RT resistance in cancer (18, 31–33). Consequently, both mTOR inhibitors and survivin inhibitors have gained significant attention in recent years due to their potential role as radiosensitizers in cancer treatment. Several clinical trials have explored the combination of mTOR inhibitors with radiation therapy in various cancer types (19–23). These trials mostly aimed to assess the safety and efficacy of this combination strategy and findings from these studies suggest potential benefits. Based on the above observations, we hypothesized that simultaneously inhibiting these two pathways would augment the effect of radiation on cancer cells synergistically. Indeed, the clonogenic assay in our study showed a moderate-to-strong synergistic effect of this combination in two different RCC cell lines (Fig. 3). The combination also efficiently reduced the expressions of multiple DNA damage response elements (Fig. 3). Hence, it is not a surprise when the combination augmented the effects of radiation in a subcutaneous RCC xenograft model (Fig. 4).

However, this xenograft model does not consider the effect of an intact immune system on the outcome of RT. RT not only exerts cytotoxic effects on tumor cells but also amplifies antitumor immunity by modifying the tumor microenvironment (TME) to elicit a potent antitumorigenic immune response (48–51). RT induces immunogenic cell death, resulting in the release of various cytokines and chemokines into the TME, which serve as chemoattractants facilitating the infiltration of dendritic cells (DCs) to the tumor site (52). The activation of DCs and the upregulation of cytotoxic T lymphocytes are believed to be the cause of the radiation-induced antitumorigenic immune response (53, 54). Conversely, RT has demonstrated the ability to induce immunosuppression by promoting the infiltration of regulatory T cells (Tregs) and myeloid-derived suppressor cells (MDSCs) into the TME (55–57).

Everolimus, typically immunosuppressive, has been shown to increase the abundance of Tregs and MDSCs in both the TME and circulation (58). Although the tumor-targeted liposomal formulation is anticipated to reduce the systemic exposure of everolimus, its potential to elevate immunosuppressive Tregs and MDSCs in the tumor microenvironment, thereby counteracting any immune-mediated enhancement of radiation therapy, cannot be disregarded. On the contrary, survivin, released from cancer cells into the TME, serves as a modulator of the T cell response, inhibiting their proliferation and inducing a shift to a type 2 response (59). Therefore, the presence of the survivin inhibitor YM155 in EY-L is expected to mitigate the immunosuppressive effect of everolimus to some extent. Indeed, our data suggests that EY-L treatment, either alone or in combination with radiation, demonstrated slightly increased CD8 + T cell infiltration in Renca tumors (Fig. 5), which may be responsible for a comparatively better antitumor response for EY-L + R treatment in Renca tumors than 786-O tumors.

Mitotic catastrophe is considered a form of cell death that occurs during or after abnormal mitosis. It is an important aspect of the cellular response to DNA damage, including damage induced by radiation (45). When this damage is severe and beyond repair, the cell may undergo mitotic catastrophe as a response. Typically, cells have mechanisms to halt the cell cycle to allow for repair in response to DNA damage. If the damage is extensive and irreparable, cells may be arrested in the G2 phase of the cell cycle. Despite the cell cycle arrest, some cells may attempt to undergo mitosis. This is problematic because the damaged DNA is often unevenly distributed between the daughter cells, leading to genomic instability. This can result in cell death or the generation of cells with abnormal chromosome numbers and structures. Mitotic catastrophe often triggers programmed cell death pathways, such as apoptosis or necrosis, as a protective mechanism to eliminate cells with severely damaged DNA and prevent the propagation of genetic abnormalities (60). This has led cancer researchers across the globe to exploit mitotic catastrophe as an attractive avenue for cancer therapy (61).

Interestingly, survivin participates in the chromosomal passenger complex and ensures accurate separation of sister chromatids and microtubule stabilization at the late stages of mitosis (62). Consequently, loss-of-function of the gene encoding survivin can lead to mitotic disturbances such as mitosis delay, chromosome displacement, and cell accumulation in prometaphase (63). RNAi-based survivin knockdown has been previously shown to induce mitotic catastrophe in multiple cancer and non-cancer cell lines (64–67). Additionally, Y-L downregulates Chk1 and Chk2, both of which are negative regulators of mitotic catastrophe (68, 69). On the other hand, mTOR inhibitors alone are not known to induce mitotic catastrophe but a few studies have shown that a combination of mTOR inhibitors with other genotoxic agents such as Chk1 inhibitor and HASPIN inhibitor were able to induce mitotic catastrophe in cancer cells (70, 71). Since YM155 (as Y-L) inhibits Chk1 (Fig. 3), it is plausible that a combination of everolimus with YM155 would do the same. Indeed, our data shows that EY-L, especially in combination with radiation, induced mitotic catastrophe in RCC tumors in vivo, as illustrated by the abundance of multinucleated cells in the H&E-stained tumor sections (Fig. 5) and a significant increase in pericentrin/nuclei ratio (Fig. 6).

## CONCLUSION

In summary, our study utilized a rational combination of an mTOR inhibitor and a survivin inhibitor in a tumor-targeted liposomal formulation to augment radiation therapy in renal cancer by inhibiting DNA damage repair and enhancing mitotic catastrophe. The combination itself showed excellent tumor growth inhibition, so, the proposed strategy is poised to act through a two-pronged assault on cancer cells: a) directly affecting tumor growth and b) sensitizing cancer cells toward radiation. While the present study is focused on renal cancer, this strategy may also be useful in other cancer indications since both everolimus and YM155 have been shown to act as radiosensitizers in a variety of cancers including lung cancer, breast cancer, prostate cancer, and glioblastoma.

## Data Availability

All data generated or analyzed during this study are included in this published article and its supplementary information files.
